# Flutter sensitivity in FM bats. Part I: delay modulation

**DOI:** 10.1007/s00359-018-1291-z

**Published:** 2018-09-22

**Authors:** A. Leonie Baier, Lutz Wiegrebe

**Affiliations:** 1 Department Biology II, Ludwig Maximilians University Munich, Großhaderner Str. 2, 82152 Martinsried, Germany; 2 Acoustic and Functional Ecology Group, Max Planck Institute for Ornithology, Eberhard-Gwinner-Str. 11, 82319 Seewiesen, Germany

**Keywords:** Biosonar, Echolocation, Virtual target, Doppler, Wagon-wheel effect

## Abstract

Echolocating bats measure target distance by the time delay between call and echo. Target movement such as the flutter of insect wings induces delay modulations. Perception of delay modulations has been studied extensively in bats, but only concerning how well bats discriminate flutter frequencies, never with regard to flutter magnitude. We used an auditory virtual reality approach to generate changes in echo delay that were independent of call repetition rate, mimicking fluttering insect wings. We show that in the frequency-modulating (FM) bat *Phyllostomus discolor*, the sensitivity for modulations in echo delay depends on the rate of the modulation, with bats being most sensitive at modulation rates below 20 Hz and above 50 Hz. The very short duration of their calls compels FM bats to evaluate slow modulations (< about 100 Hz) across entire echo sequences. This makes them susceptible to interference between their own call repetition rate and the modulation rate. We propose that this phenomenon constitutes an echo-acoustic wagon-wheel effect. We further demonstrate how at high modulation rates, flutter sensitivity could be rescued by using spectral and temporal cues introduced by Doppler distortions. Thus, Doppler distortions may play a crucial role in flutter sensitivity in the hundreds of FM species worldwide.

**Electronic supplementary material:**

The online version of this article (10.1007/s00359-018-1291-z) contains supplementary material, which is available to authorized users.

## Introduction

Bats use echolocation to detect targets such as insect prey. They emit ultrasonic calls that are reflected off a target and return to the bat as echoes carrying information about the target. Two general types of echolocation calls have evolved in bats: frequency-modulated calls (FM calls) sweep through a broad band of frequencies within a few milliseconds, whereas constant-frequency calls (CF calls) keep a constant frequency over a much longer duration. The spatial acuity at which a target is localized increases with the range of frequencies an echolocation call covers, the call bandwidth. Acuity in target-distance assessment directly depends on bandwidth (Simmons 1973; Siemers and Schnitzler 2004), and acuity in azimuth and elevation indirectly depends on bandwidth, because broadband calls typically contain higher frequencies that give better spatial acuity due to shorter wavelength and higher directionality (Griffin 1958). The temporal resolution, at which changes in a target are depicted, however, is limited by the duration of the call (for, e.g., Doppler shift-based analyses) and/or call repetition rate (for time-domain analyses of echo-delay variation). A broadband, short FM call emitted at relatively low duty cycles therefore grants high spatial acuity at the possible expense of accuracy in detecting the movement of the target. Note that frequency-modulating bats (FM bats) constitute more than 80% of all echolocating species (Nowak 1994) and are able to navigate and forage in an environment full of moving targets.

Echolocation is a trinity of call, target and echo. Both call properties and target properties determine the properties of the echo. Knowledge of the call properties and the echo properties in turn lets the bat draw conclusions about the target properties such as its location or surface structure (Simmons et al. 1974, 1983; Lawrence and Simmons 1982; Schmidt 1988b; Weissenbacher and Wiegrebe 2003; Grunwald et al. 2004; Holderied and von Helversen 2006; Firzlaff et al. 2007; Falk et al. 2011). For instance, the distance to the target is directly encoded in the time it takes the sound to travel from the bat to the target and back: the echo delay. Another parameter that changes with target distance is the amplitude of the returning echo. The further the sound travels, the fainter it becomes. Additionally, echo amplitude depends on the reflective strength of the target, the so-called target strength (Simmons et al. 2014). In other words, echo delay and echo amplitude co-vary with distance to the target, but echo delay is an absolute cue and echo amplitude is a relative cue for target distance.

Movement of the target itself can change its distance to the bat and its target strength. Thus, movement of the target introduces changes in echo delay and echo amplitude, which is referred to as echo-delay modulation and echo-amplitude modulation, respectively. For example, the fluttering wings of insects move back and forth, thereby changing the distance of the reflecting wing area. At the same time, the wings rotate, i.e., they change the size of the reflecting wing area, thereby changing the target strength (Griffin 1958; Roeder 1963). Consequently, periodic modulations in either echo delay or echo amplitude indicate fluttering insects. The ability to detect modulations of echo parameters is therefore often referred to as flutter sensitivity.

While there is a large body of literature regarding the basics of flutter sensitivity in CF bats (reviewed in Neuweiler 1990), flutter sensitivity in FM bats has been addressed by a mere handful of studies. The behavioral studies by Sum and Menne (1988), Roverud et al. (1991) and Grossetete and Moss (1998) have invited further inquiries. Flutter sensitivity was investigated only in terms of discriminating one flutter rate from another, not in terms of absolute sensitivity to the magnitude of the flutter, i.e., how large a flutter needs to be at a given flutter rate so that it can be detected by the bat. Moreover, neither study independently assessed bats’ sensitivity to the two types of modulation introduced by the flutter: the modulation of echo delay and the modulation of echo amplitude. In both studies, the echolocating bat was presented with real targets, where echo delay and echo amplitude co-vary and it is therefore impossible to elucidate which information the bats extract from delay versus amplitude modulations. The key to solving this question is to create an auditory virtual reality for a bat and present virtual targets where amplitude and delay can be independently controlled.

A virtual target is communicated to the bat by a computer-generated echo played from a loudspeaker. Virtual targets produce simulated reflections, generated by picking up the bat’s emission with an ultrasonic microphone, convolving it in real time with the acoustic impulse response of the virtual target, and playing back the result as an echo with a short latency of only a few milliseconds. The impulse response is the acoustic image of a target. It consists of the sum of all acoustic reflections of a target when it is ensonified with an acoustic impulse.

The classical phantom-target jitter experiments by Simmons (1979) that were repeated by Menne et al. (1989) took advantage of this method to selectively modulate only the echo delay and examine sensitivity to the magnitude of delay changes. However, these experiments were not designed to assess flutter sensitivity and therefore the modulation rate was not studied as an independent parameter: the rate of the rectangular echo-delay modulation was determined by the rate of sonar emissions; the phantom target ‘jumped’ back and forth with every emitted call. Notably, this is not an ecologically plausible modulation: in these experiments, target properties were adjusted according to the bat’s vocal behavior, while in natural situations it is the reverse, i.e., bats adjust their ensonification behavior according to target properties (Moss and Surlykke 2010). Only one study has investigated bats’ sensitivity to the magnitude of echo-delay modulation for a fixed, call-independent jitter frequency: Goerlitz et al. (2010) trained free-flying bats to discriminate between a stationary loudspeaker membrane and a membrane sinusoidally vibrating at 10 Hz. The perceived call-to-call jitter depended on call rate and call emission time in relation to the modulation phase. Thus, we hypothesize that for evaluation of changes across entire sequences of call–echo pairs the relation between call rate and modulation rate plays a crucial role.

To test this hypothesis, we combined both approaches, the sinusoidal modulation of either delay or amplitude independently from the bat’s emission rate. This relies on virtual targets that change over time in just one of the two parameters, delay and amplitude. With modern processors that can operate in real time at high sampling rates, we can use time-variant impulse responses to create such virtual targets. The important advantage of a time-variant impulse response is that it truly simulates a moving target: it produces an echo with the target properties at the specific moment in time when the call is emitted, so that it interacts with call properties such as call duration and inter-call interval, affecting echo frequency, duration and delay. Like real moving targets, time-variant impulse responses can thus create, e.g., Doppler distortions and echo-amplitude modulations.

In this two-part study, we used a virtual environment to manipulate first only the modulation of delay at many call-independent modulation rates and second only the modulation of amplitude at many call-independent modulation rates. In this first of a series of two papers, we report on our investigations of the first part: bats’ sensitivity to delay modulation. We demonstrate that sensitivity to echo-delay modulation strongly depends on modulation rate: bats show good sensitivity at low and high modulation rates and worse sensitivity for intermediate modulation rates around 20 and 50 Hz.

## Materials and methods

### Animals and permit

We used six adult male individuals of the neotropical omnivorous bat species *Phyllostomus discolor*, Wagner, 1843.These bats emit short (< 3 ms), downward frequency-modulated, multi-harmonic echolocation calls covering the frequency range between 45 and 100 kHz (Rother and Schmidt 1982). Bats were kept at the bat facilities in the Department Biology II of the Ludwig-Maximilians-University in Munich (12 h night/12 h day cycle, 65–75% relative humidity, 28 °C) with unlimited access to water at all times. On free days, the bats had ad libitum access to mixed fruit and mealworms (larval form of *Tenebrio molitor*) supplemented with oat, safflower oil, baby formula, minerals and vitamins (Vitakalk^®^). During training periods, the bats were with fed a pulp from fruit and supplementals in the experiment. All experiments complied with the principles of laboratory animal care and were conducted under the regulations of the current version of the German Law on Animal Protection (approval 55.2-1-54-2532-34-2015, Regierung von Oberbayern).

### Experimental setup

**Fig. 1:**
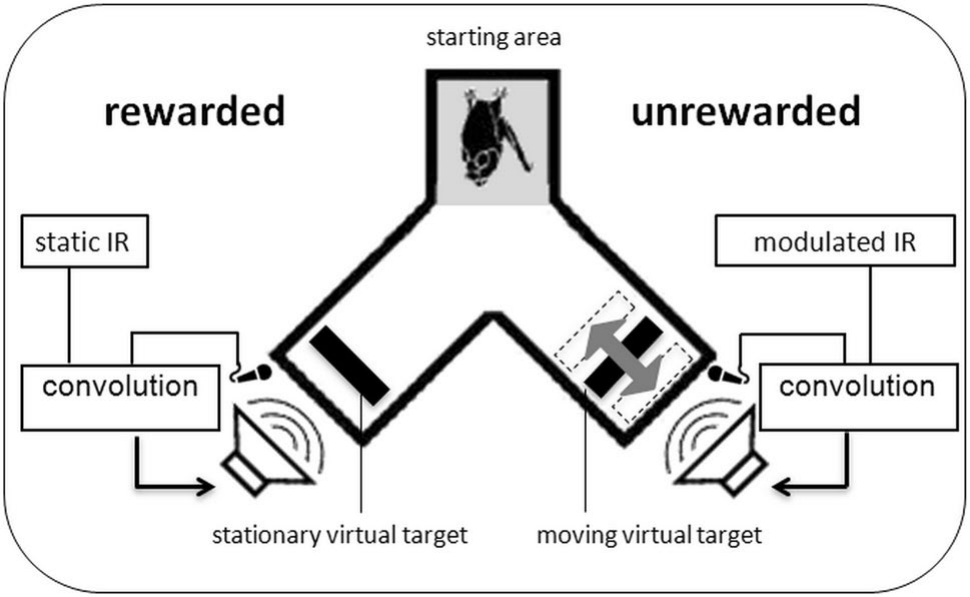
Auditory virtual reality setup: six bats were trained to discriminate a virtual stationary target from a virtual target that simulated a periodic movement through modulating the echo delays at varying modulation rates ranging from 2 to 1000 Hz. All bats learned to indicate the pseudorandomly chosen position of the stationary target by crawling toward it from the depicted starting area after echolocating toward both targets. Virtual targets were created by convolving recorded echolocation calls in real time with a static or time-variant impulse response (IR)

Bats were trained to discriminate a rewarded stationary virtual target from an unrewarded target whose delay was sinusoidally modulated. The experiments were performed in a Y-maze inside a dark echo-attenuated chamber. The Y-maze consisted of a wire mesh floor, covered with removable cloth to clean the setup; the walls and ceiling of the maze were made of acoustically transparent gauze suspended between thin (about 2 mm diameter) metal rods at the corners of the maze. The starting area of the maze (lightly shaded area in Fig. 1) was about 10 cm wide and 15 cm long; each leg of the maze was also 10 cm wide and about 20 cm long. The inner height of the gauze was 12 cm throughout. As illustrated in Fig. 1, the loudspeakers and microphones were mounted directly behind the acoustically transparent gauze at the end of the two legs of the Y-maze. The experimenter was stationed outside the chamber and observed the experiment via an infrared camera (Abus^®^ TV6819) and headphones. Stimulus presentation and data recording were controlled via a custom MatLab^®^ R2007b application (The Mathworks, Inc., Natick, MA, USA).

### Virtual-target production

During the full length of a trial, a bat would utter echolocation calls, and we implemented a time-variant delay between the call recording through the microphones and the virtual echo playback via the loudspeakers (henceforth, we will refer to the virtual echoes simply as echoes, although they were not echoes in the strict sense of an echo being a reflection from a physically present surface). Virtual targets were otherwise implemented as simple reflectors. Every change the bat chose to make in its emission sequence (e.g., change in call timing or call spectrum) was immediately reflected in the echoes. The only parameter that was systematically varied on our part was the echo delay.

Specifically, the bat’s ultrasonic emissions were picked up by two microphones (SPM0204uD5, Knowles Corporation, Itasca, IL, USA) mounted 45° left and right relative to the bat’s starting perch in a Y-maze. The microphone signals were amplified (octopre LE, Focusrite plc, Bucks, UK) and fed into the inputs of a real-time digital signal processor (260 kHz sampling rate; RX6, Tucker Davis Technologies, Gainesville, FL, USA). In the processor, a dynamic delay component, driven by a sine-wave generator of adjustable amplitude and frequency, was used in the modulated target’s channel before the inputs were routed to the outputs, in addition to a constant base delay of 2500 µs in both channels. Together with the AD, DA sampling delays of the RX6 and the physical delays from the bat to the microphone and from the speaker to the bat, the overall echo delay (without modulation) was about 4200 µs. This means that the virtual target was presented at a virtual distance of 72 cm to the emitting bat. It “appeared” outside the physical setup so that the bat could separate physical from virtual echoes more easily. Feedback suppression circuitry was included for safe operation. The outputs were connected via a stereo amplifier (Harman Kardon HK 6150; Harman Deutschland, Heilbronn, Germany) to two ultrasonic speakers (Technics EAS10TH800D; Panasonic Deutschland, Hamburg, Germany). Bats were tested with modulation depths of ± 2048 µs, ± 1024 µs, ± 512 µs, ± 128 µs, ± 64 µs, ± 32 µs, ± 16 µs and ± 8 µs for each of the following modulation rates: 2, 5, 10, 20, 50, 100, 200, 500, and 1000 Hz. At a modulation rate of 2 Hz, the signal undergoes one full modulation period within 500 ms, i.e., from original delay to shorter delay, to original delay, to longer delay, and finally to original delay again. At a modulation depth of ± 2048 µs, the virtual target moves within one modulation period from the reference distance of 72 cm (4200 µs) over a distance of 35 cm to the front, then again to the reference distance, then over a distance of 35 cm to the back, and finally back to the reference distance.

### Behavioral procedure

Training/recording sessions (one to three per day) each lasted 10 min. Bats were trained on 5 days per week, followed by a 2-day break. The experiment followed a two-alternative, forced-choice paradigm (2AFC) with food reinforcement. Once a bat sat or perched in the starting area of the Y-maze, presentation of the virtual targets was switched on. The position of the stationary target (left or right) was pseudorandom (Gellermann 1933). Bats had to echolocate to find and move toward the stationary target, where they were rewarded as soon as they reached the corresponding feeder. Once a bat had learned this task (> 70% correct choices on 5 consecutive days), the modulation depth of the unrewarded target was reduced, making the discrimination task more difficult. During data acquisition, the modulation depth was then further reduced, starting with three consecutive trials presenting the highest modulation depth of ± 2048 µs, stepwise going down to three trials at a modulation depth of ± 8 µs and starting at ± 2048 µs again, etc. until the daily sessions were completed. To keep the bats motivated, easier trials (with a larger modulation depth) could be interspersed. Testing for one modulation rate set was completed when at least 30 trials were obtained per modulation depth and bat.

### Behavioral data analysis

**Fig. 2:**
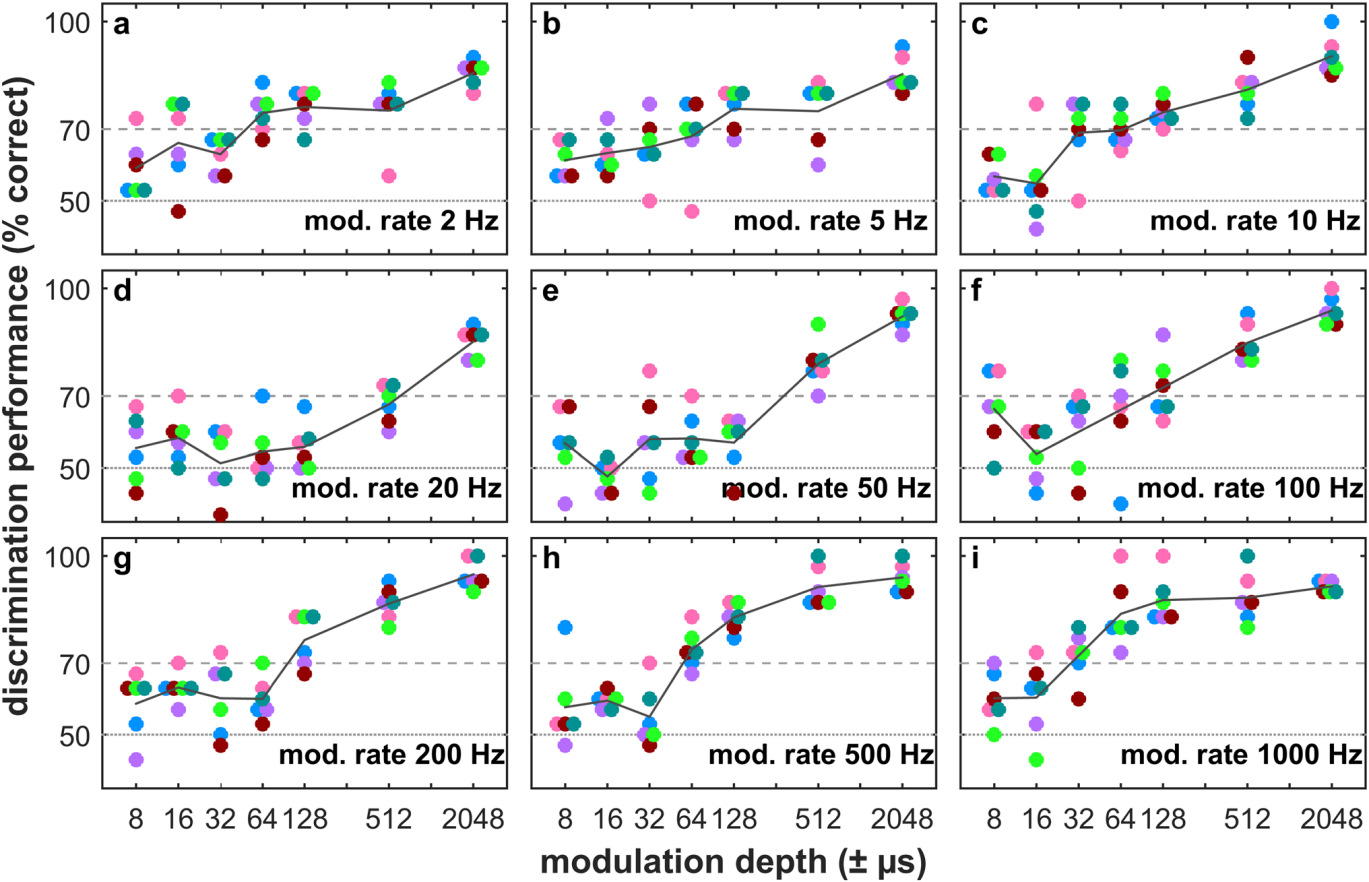
Psychometric functions of echo-delay-modulation discrimination performance at nine modulation rates. Each colored dot marks one bat’s discrimination performance across 30 trials. Black line plots depict the average discrimination performance. Horizontal dashed lines at 50 and 70% correct depict chance and significance level, respectively

Percent correct performance of the animals as a function of modulation depth was fitted with a sigmoidal function and the value of this fit at 70% was taken as threshold (for *p* < 0.05 in a binomial test cf. Fig. 2). The threshold is the just-noticeable difference (JND) in modulation for a single bat at a single modulation rate.

### Acoustic analyses

The echo properties depended both on the properties of the virtual targets themselves and critically on the properties of the emitted calls that the bats used to ensonify them. In our study, we manipulated the echo-acoustic target properties. We verified the echolocation-call properties with sound analysis. Additionally we verified the resulting echo properties generated by our delay-modulation hardware (the “virtual target machine”) in response to an artificial echolocation call (see below). All acoustic analyses were done with custom MatLab^®^ R2015a programs.

For sound analysis, the recorded call sequences were saved in a 3-s stereo ring buffer (192 kHz sampling rate, 24-bit resolution; Motu Ultralite, Motu, Cambridge, MA, USA) parallel to the virtual-target production. We high-pass filtered the stereo recordings at 35 kHz applying an eighth-order Butterworth filter. Then we extracted all echolocation calls above a fixed detection threshold (− 46 dB re. full scale) and with a minimum spacing of 5 ms between subsequent signals to exclude potentially recorded echoes. Temporal and spectral call parameters were taken from the channel with higher call level. We calculated the inter-call interval and the − 10 dB call duration. Call levels were calculated across a fixed 2.5 ms window centered on each call. The spectral centroid (weighted mean of frequencies present in the signal) was calculated from a time-averaged spectrogram with a 750 Hz binwidth. Minimum and maximum frequencies were extracted 10 dB below the peak frequency.

For the measurements of echo properties, we generated an artificial echolocation call as a multiharmonic FM-downward sweep of 1 ms duration with a fundamental frequency ranging from 23 to 19 kHz. This artificial echolocation call was then fed into the delay-modulation hardware (RX6; Tucker Davis) and manipulated in the same way as the real echolocation calls during the experiment. The signal at the processor’s output was saved as the artificial echo, either from the stationary reflector or from the modulated reflector. For the latter, the outcome depended on the modulator phase that the sweep interacted with. We thus analyzed a coherent subset of echoes created at eight equally spaced phases in steps of 45° during the modulation.

## Results

### Behavioral response

Six male FM bats (*Phyllostomus discolor*) learned to discriminate between a virtual echo presented at a constant delay and a virtual echo presented at a modulated delay. We used the behavioral response of the bats to assess the just noticeable modulation depth, i.e., the threshold. For every bat, we extracted one threshold per modulation rate from the psychometric function to form a modulation transfer function across the nine modulation rates. It describes the sensitivity of the FM bat *P. discolor* for the modulation of echo delay.

Across all modulation rates, the results of all six bats confirmed our expectations for a psychometric function: discrimination was good at large echo-delay modulation depths and deteriorated with decreasing modulation depth (Fig. 2).

All bats faithfully (80–100% correct choices) discriminated a stationary target at a delay of 4200 µs from a target that oscillates in delay by ± 2048 µs around 4200 µs. On the contrary, the most difficult discrimination task we set, with a modulated target oscillating in delay by ± 8 µs around 4200 µs, could not be solved at all (40–67% correct choices for five of the nine tested modulation rates; Fig. 2b–e, g), or was only solved by one or two bats (70–80% correct choices for four modulation rates; Fig. 2a, f, h, i).

Discrimination performance systematically changed with the rate of the echo-delay modulation. Starting at the lowest modulation rate of 2 Hz, where the average discrimination threshold lies between ± 32 and ± 64 µs delay modulation, the bats’ average performance deteriorates with increase in modulation rate up to a modulation rate of 20 Hz, where the average discrimination threshold lies between ± 512 and ± 2048 µs delay modulation. When the modulation rate is further increased up to 1000 Hz, bats’ performance monotonically improves again (average discrimination threshold between ± 32 and ± 64 µs delay modulation).

**Fig. 3:**
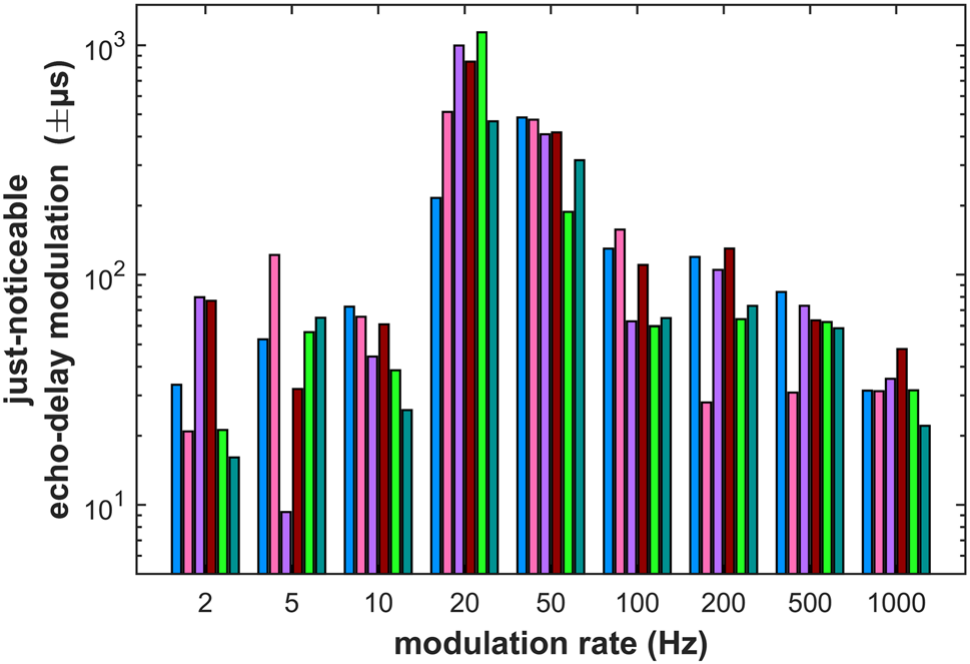
Echo-delay modulation sensitivity. Detection thresholds are generally best at very low and very high modulation rates, and worst at intermediate modulation rates of 20 Hz and 50 Hz. Note the logarithmic ordinate. Bar colors represent individual bats’ thresholds as extracted from sigmoidal fits to the psychometric functions in Fig. 2. Also note that modulation thresholds are given as peak values; they can be converted to peak-to-peak thresholds by multiplication with two

The just-noticeable difference in modulation (JND) values extracted from the nine psychometric functions form the modulation transfer function that describes the bats’ sensitivity for echo-delay modulation across nine modulation rates (Fig. 3). The modulation transfer function shows that the bats perform well at low and high modulation rates with echo-delay JNDs better than ± 100 µs. However, for intermediate modulation rates of 20 or 50 Hz, JNDs deteriorate and the bats need around ± 400 to ± 700 µs delay modulation to discriminate the stationary target from the modulated one.

### Acoustic analyses

The bats’ auditory percept depended not only on the echo-acoustic features of the virtual targets themselves, but critically on how the bats ensonified them. We performed acoustic analyses of the echolocation calls used by the bats during the behavioral experiment to better understand which sensory- and vocal-motor strategies the bats employed to solve the task. Additionally, we measured the echo properties generated by our delay-modulation hardware in response to an artificial echolocation call.

**Fig. 4:**
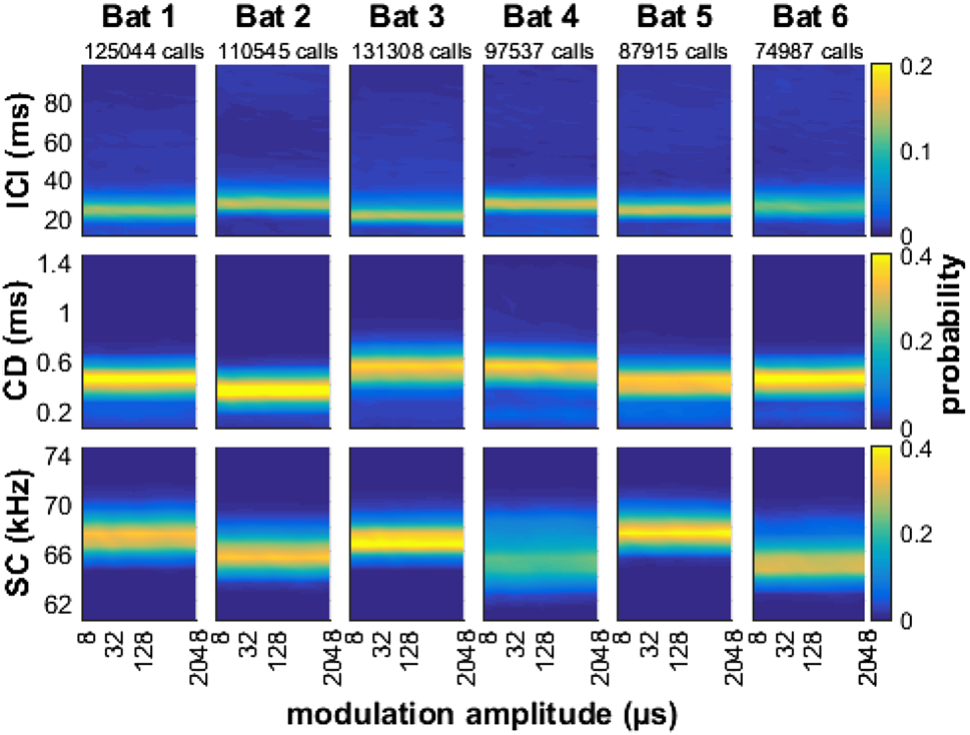
Temporal and spectral properties of echolocation calls used by the bats for detecting echo-delay modulations with different modulation depths. The distribution of inter-call intervals (ICI, Row 1), call durations (CD, Row 2) and spectral centroids (SC, Row 3) did not change as a function of modulation depth (i.e., task difficulty) in either of the six bats (columns). Data are shown as normalized bin counts with color-coded probability

**Fig. 5:**
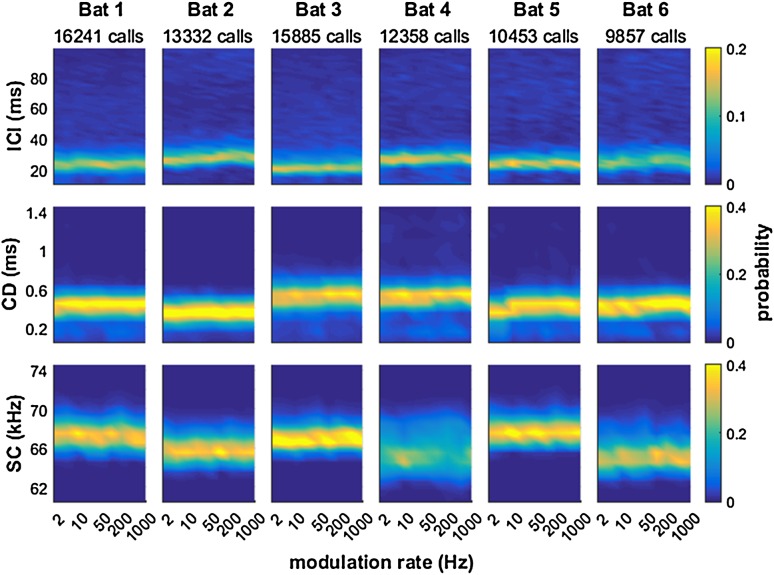
Temporal and spectral properties of echolocation calls used by the bats for detecting echo-delay modulations with different modulation rates at a modulation depth that was just detectable for the bats. Again, the distribution of inter-call intervals (ICI, Row 1), call durations (CD, Row 2) and spectral centroids (SC, Row 3) did not change systematically as a function of presented modulation rate in the six bats. Again, data are shown as normalized bin counts with color-coded probability

In the acoustic analysis of the echolocation calls, we first tested whether fundamental call parameters like inter-call intervals (ICIs), call duration, or the spectral centroid of the calls changed systematically when the task became more difficult for the bats, i.e., when the modulation depth decreased. The data show that, referenced against data from the highest modulation depth, the bats did not systematically modify any of these call parameters with increasing task difficulty (Fig. 4). Second, we determined whether these call parameters changed systematically with modulation rate. Here, we used only data from those trials where modulation depth was close to the perceptual threshold for this modulation rate and bat. The data show that ensonification parameters of the bats remain different across bats, but rather constant as a function of modulation rate (Fig. 5). In conclusion, we found no evidence for an adjustment of ensonification parameters, i.e., on the vocal-motor side of echolocation,  that may serve to explain the dependence of echo-delay JNDs on modulation rate.

During the analysis of the echolocation calls, it was conspicuous that the dominant ICI across all bats was around 20–40 ms (cf. first row of Figs. 4, 5). This typical ICI was the ICI used by the bats within call groups. The psychophysical results show that performance of the bats was worst around modulation rates of 20–50 Hz. This corresponded to modulation periods of 50 and 20 ms, respectively. We conclude that the bats performed worst when their call repetition rate was similar to the modulation rate and propose an echo-acoustic version of the visual wagon-wheel effect.

**Fig. 6:**
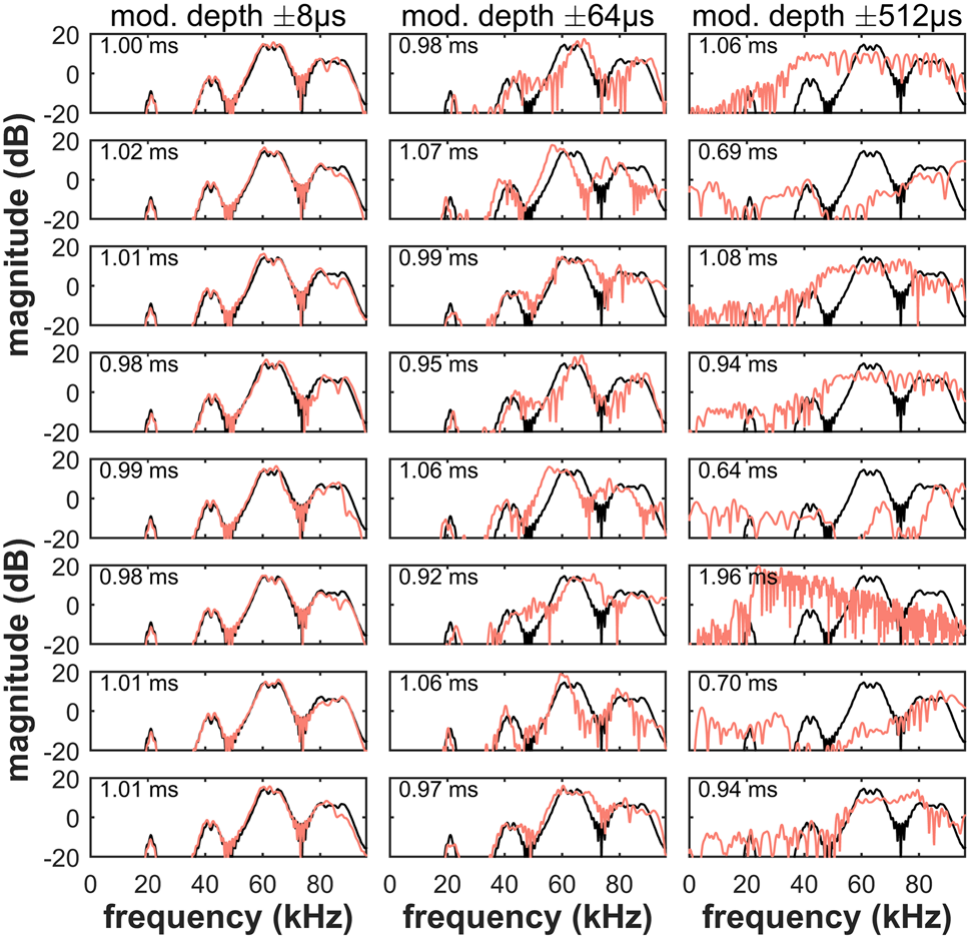
Power spectra of echoes from eight different phases (rows) of a reflector that changes in echo delay at a rate of 200 Hz (red) in comparison to the echo from the stationary reflector (black). Depth of the echo-delay modulation is either non-detectable (± 8 µs, left column), close to threshold (± 64 µs, central column) or well above threshold (± 512 µs, right column). With increasing modulation depth, the differences between echoes from the modulated reflector (red) and the stationary reflector’s echo (black) become more and more pronounced. In addition to the changes in the spectral properties, the Doppler-type distortions also introduce variation in the duration (numbers in panels) of the modulated reflector’s echoes. The duration of the stationary echo is 1 ms

For the measurements of echo properties, we used a stereotyped *P. discolor* echolocation call, a 1 ms multiharmonic FM-downward sweep, and analyzed the artificial echoes as they were created by the delay-modulation hardware. We compared echo-power spectra and duration of echoes from the stationary reflector (black) and the modulated reflector (red), each for eight different modulator phases (Fig. 6). Delay-modulation depths had been adjusted such that the modulation was not detectable (± 8 µs), close to threshold (± 64 µs) or well above threshold (± 512 µs). With increasing modulation depth, the echo-delay modulation introduced Doppler-type distortions. The echoes from the modulated reflector differed from the stationary reflector’s echo mainly in two ways: first, the frequency content was altered; second, the echo from the modulated reflector was either stretched or compressed in time relative to the echo from the stationary reflector. While these changes in echo spectrum and echo duration were moderate close to the threshold, distortions were dramatic for high modulation depths (± 512 µs). The range of frequencies below 35 kHz, which did not contain much energy in the echo from the stationary reflector, did contain energy in the echoes from the modulated reflector. Also, echo duration (numbers in panels) varied considerably between 0.64 and 1.96 ms (relative to the 1 ms call duration). Note that with the echo delay changing by ± 512 µs at a rate of 200 Hz, the virtual target would move back and forth at an average velocity of about 70 m/s, i.e., 250 km/h. These results illustrate that on top of the nominal perceptual cue, the time-variant echo delay, Doppler-type distortions may provide both spectral and temporal (echo duration) cues that may allow the bat to discriminate between stationary and time-variant reflectors.

## Discussion

When the distance between an echolocating bat and its target changes, the delay and amplitude of the echo change together, or co-vary. We found that the sensitivity of FM bats for modulations in echo delay depend on the rate of the modulation: *Phyllostomus discolor* bats were well able to distinguish a virtual target with constant echo delay from a virtual target whose echo delay was modulated over time when the modulation rates were either below 20 Hz or above 50 Hz. To the best of our knowledge, this study presents the first evidence of an echolocating FM bat detecting a delay modulation across a wide range of modulation rates that are independent of the bat’s own emission rate.

In the following paragraphs, we first discuss delay-modulation sensitivity results in the context of previous work. Second, we address the bats’ acoustic signals. Third, we propose an echo-acoustic wagon-wheel effect and discuss its origins and consequences. Fourth, we examine how fast target movements can induce alterations in both echo spectral composition and echo duration, which may serve as additional perceptual cues. Lastly, we discuss our results in an ecological context.

### Delay-modulation sensitivity

In the following, we will compare the current results to those from both delay-discrimination and delay-jitter experiments. For convenience and comparability, we will convert all thresholds into peak-to-peak thresholds in microseconds. Our *P. discolor* bats were very sensitive to low and high modulation rates: for modulation rates below 20 Hz and above 50 Hz, we found delay-modulation thresholds between 66 and 194 µs (Fig. 3). These results are similar to the results from delay- (= range) discrimination experiments, which yielded thresholds between 36 and 176 µs (Simmons 1973; Roverud and Grinnell 1985; Surlykke and Miller 1985; Masters and Jacobs 1989; Miller 1991; Denzinger and Schnitzler 1994; Masters and Raver 1996).

At low modulation rates between 2 and 10 Hz, the current detection thresholds are also comparable to the delay-modulation thresholds of *Glossophaga soricina* FM bats: Goerlitz et al. (2010) measured delay-modulation thresholds of around 73 µs for large real targets moving at a 10 Hz modulation rate. At 10 Hz modulation rate, our bats on average detected a modulation of about 103 µs depth.

To intermediate modulation rates of 20 Hz and 50 Hz, our bats were much less sensitive, with thresholds around 1340 µs and 760 µs, respectively (Fig. 3). Heinrich and Wiegrebe (2013) showed that *P. discolor* bats can just discriminate stationary virtual targets when they differ in echo delay by around 250 µs. Current modulation thresholds are worse than 250 µs for modulation rates of 20 and 50 Hz but better than 250 µs for modulation rates above or below this range. This comparison indicates that our bats may have encountered special difficulties in detecting delay modulations for modulation rates around 20–50 Hz. This will be discussed in detail below.

The current thresholds and those we have compared them to so far (from delay-discrimination experiments) are worse than thresholds in delay-jitter experiments by orders of magnitude (Simmons 1979; Simmons et al. 1990, 2003, 2004; Menne et al. 1989; Moss and Schnitzler 1989). In those experiments, delay-jitter thresholds were always below 1 µs. It appears difficult to reconcile these diverging data sets. First, it is conceivable that this divergence results from the different bat species. However, delay-discrimination thresholds in *Eptesicus fuscus* were also much worse than the sub-microsecond thresholds reported in the jitter experiments in the same species (Denzinger and Schnitzler 1994, 1998). Second, the cause for the divergence may lie in the different virtual target stimulation: in the jitter experiments, delay was switched, in a quasi rectangular manner, after each emission of the bat. Thus the bat itself determined the average modulation rate, which is half the emission rate (and of course non-periodic due to the non-periodic emission patterns). In contrast, both (Goerlitz et al. 2010) and the current data were obtained with a sinusoidal modulator, completely independent of the bat’s emission rate. Finally, differences between delay-jitter experiments on the one hand and delay-discrimination experiments on the other hand may lie in the fact that for the former, the bat can detect a change in delay at the same point in space (azimuth and elevation), while for the latter, the bat must compare delays across different azimuths. In summary, the current data remain hard to reconcile with the hyperacuity results in *E. fuscus*.

### Acoustic analysis of echolocation calls

During target approach, bats systematically decrease both inter-call intervals (ICIs) and call duration to prevent the returning echo from overlapping with their next call (Griffin et al. 1960). For *P. discolor*, Linnenschmidt and Wiegrebe (2016) also observed that when a food source approached the bats, they systematically decreased the ICIs, the call duration and also the sound level of their emissions. In the current data, however, such an adjustment of call parameters is not seen (Figs. 4, 5): call parameters do not change, neither as a function of the delay-modulation depth (task difficulty) nor as a function of delay-modulation rate. Note that in the current experiments, there was no linear target motion, but target distance changed sinusoidally around a constant reference distance of 72 cm.

In the experiments of Linnenschmidt and Wiegrebe (2016), *P. discolor* use call durations of 0.4–0.7 ms and ICIs of 25–50 ms when echolocating toward a target at 70–80 cm distance. The call durations and ICIs we found in the current experiment (call duration around 0.4 ms and ICIs around 29 ms) are a good match given the here simulated distance of 72 cm between the virtual target and the bat. When we assume that perceived target distance dictates the call parameters that bats employ also in a stationary situation, we can explain why they neither adjusted their emissions to modulation depth nor to modulation rate. At the same time, this raises the question how this quasi-stable ICI interacts with the echo-delay modulation. In the following paragraph, we address our proposition that this represents an echo-acoustic version of the visual wagon-wheel effect.

### Echo-acoustic wagon-wheel effect

We hypothesize that an interference of the ICI with the rate of the modulation generates an echo-acoustic wagon-wheel effect. In vision, the wagon-wheel effect is the result of temporal aliasing and describes the effect that under stroboscopic illumination a periodic movement may stay undetected because the illumination always occurs at the same phase of the movement. Transferred to the echo-acoustic system of bats, the unrewarded delay-modulated target may appear stationary (and thus more similar to the rewarded target) when the modulation rate of the modulated target equals the ensonification rate (= the reciprocal of the ICI) or an integer multiple thereof. Because the echoes perceived by the bat are the result of the given virtual target reflecting the echolocation call, the echo properties critically depend on the echolocation call parameters employed by the bats. We therefore would have expected the bats to adjust echolocation call parameters to task difficulty, i.e., to echo-delay modulation depth, but we found no evidence for such an adjustment (Fig. 4). Instead, the distribution of inter-call intervals (Fig. 4, Row 1), call durations (Row 2) and spectral centroids (Row 3) remained stable across modulation depths for individual bats. This finding supports the hypothesis that the bats very stereotypically adjust their ICI to target distance and do not intentionally vary the ICI to circumvent the wagon-wheel effect.

While we believe that this wagon-wheel effect may at least qualitatively explain the performance drop for intermediate modulation rates, this is not meant to suggest that the bats use the same perceptual cues to detect lower- and higher-rate modulations. At high modulation rates (corresponding to fast movements of the virtual target), other echo parameters may facilitate the psychophysical task (Beedholm and Møhl 1998). In the following paragraphs, we show that fast target movements can induce perceivable changes in both echo spectral composition and echo duration, related to Doppler distortions.

### Doppler distortions

Doppler distortions arise from a sound being emitted or reflected by a moving object. In echolocation, an approaching target will produce an echo of a higher frequency than the emission; for a retreating target, the echo frequency is lower than the emission frequency. At first sight, Doppler distortions may be difficult to detect for FM bats, because the short duration and broad bandwidth of FM calls hamper the distortions’ auditory detectability. Nevertheless, our measurements of Doppler distortions (Fig. 6) show that for higher modulation rates, Doppler distortions were prominent and perceptually relevant even at moderate modulation depths, comparable to the current perceptual thresholds. Specifically, the distortions caused the echo spectrum to spread below 35 kHz, where echoes from stationary targets were very faint. Such a pronounced difference in echo spectral composition is very likely perceived by bats (Schmidt 1988a; Weissenbacher and Wiegrebe 2003; Falk et al. 2011). Additionally, the distortions can almost halve or double the duration of the echo, depending on the modulator phase (0.64 ms and 1.96 ms relative to the 1.00 ms call duration). Again, this cue is well perceivable for bats (Schoernich and Wiegrebe 2008). Doppler distortions can even invert the spectro-temporal structure of the echolocation call. During an approach phase of a fast sinusoidal distance modulation, the downward frequency modulation of the emission can become an upward modulation in the echo.

The current results indicate that FM bats may be sensitive to Doppler distortions as generated by the wing beat of insects. It will be interesting to look in detail for the behavioral and neuronal mechanisms behind Doppler detection in terms of auditory temporal and tonotopic echo analysis. Specifically, analyses in Fig. 6 show that Doppler distortions affect both duration and spectral composition of echoes. Physiological sensitivity to echo spectral structure has been demonstrated both in *E. fuscus* (Sanderson and Simmons 2000) and *P. discolor* (Firzlaff et al. 2006; Borina et al. 2008; Heinrich et al. 2011). Also, neural sensitivity to echo duration was repeatedly demonstrated (Aubie et al. 2012; Fremouw et al. 2005). Finally, we have shown earlier that the *P. discolor* auditory cortex is quite sensitive to correlated changes in echo spectrum and duration and can even combine such features in a meaningful manner (Firzlaff et al. 2007).

### Ecological relevance

Notably, we also presented our bats with target velocities that possibly exceed the ones found in fluttering insects (Vanderplank 1950). For instance, the modulation parameters exemplified in Fig. 6 were 200 Hz and ± 512 µs, corresponding to an average target velocity of 70 m/s. However, at the perceptual threshold for this modulation rate, the virtual target moves back and forth across a distance of about 15 mm within 5 ms. This results in an average velocity around 12 m/s. This lies well within the range of insect wing tip velocities (e.g., mosquito: 3 m/s, tsetse fly: 18 m/s; Vanderplank 1950).

We know little about the extent to which *P. discolor* hunts fluttering insects. In fact, it is often considered a mainly frugivorous species. However, its diet strongly depends on its geographic distribution and on season, ranging from almost pure nectarivory to almost pure insectivory (Kwiecinski 2006). The nitrogen isotopic composition of Mexican *P.discolor* is indistinguishable from that of carnivorous and sanguivorous animals (Schondube et al. 2001). While the stomach content of (Brazilian) *P. discolor* reportedly includes many insect species capable of flight (Willig et al. 1993), we cannot know whether this prey was caught in flight or gleaned off the substrate. Though we cannot finally conclude whether *P. discolor* itself could make use of a sensitive flutter detection system for prey detection, we assume that a true aerial hawking bat species would greatly benefit from flutter sensitivity in FM echolocation. Furthermore, flutter sensitivity would be advantageous for detecting other target movements that produce periodic echo-delay changes and thereby indirectly represent prey. For instance, advancing water ripples may indicate the presence of prey to the frog-eating bat, the Phyllostomid species *Trachops cirrhosus* (Halfwerk et al. 2014).

In conclusion, our work offers valuable insights into the perception of fluttering targets by FM bats. We have introduced a virtual reality approach with time-variant targets to assess sensitivity to echo-delay modulation. We demonstrated that in the FM bat *P. discolor*, the sensitivity for modulations in echo delay depends on the rate of the modulation. Sensitivity was best at modulation rates below 20 Hz and above 50 Hz. We suggest that an echo-acoustic wagon-wheel effect diminishes delay information when the modulation rate of the target matches bats’ call repetition rate or an integer multiple thereof. We speculate that at high modulation rates, bats instead use spectral and temporal cues introduced by Doppler distortions.

The use of virtual targets allows the clean segregation of echo-delay and echo-amplitude modulations for flutter detection. The following paper will address bats’ sensitivity to echo-amplitude modulations. We will show that echo-amplitude modulation is perceived quite differently from echo-delay modulation, indicating fundamentally different neural processing of these co-occurring echo features.

## Electronic supplementary material

Below is the link to the electronic supplementary material.

Supplementary material 1 (DOCX 157 KB)

## Abbreviations

CF: Constant frequency
FM: Frequency-modulating/frequency modulation
ICI: Inter-call interval
JND: Just noticeable difference
2AFC: Two-alternative forced choice

## References

[CR1] Aubie B, Sayegh R, Faure PA (2012). Duration tuning across vertebrates. J Neurosci.

[CR2] Beedholm K, Møhl B (1998). Bat sonar: an alternative interpretation of the 10-ns jitter result. J Comp Physiol A.

[CR3] Borina F, Firzlaff U, Schuller G, Wiegrebe L (2008). Representation of echo roughness and its relationship to amplitude-modulation processing in the bat auditory midbrain. Eur J Neurosci.

[CR4] Denzinger A, Schnitzler H-U (1994). Echo SPL influences the ranging performance of the big brown bat, *Eptesicus fuscus*. J Comp Physiol A.

[CR5] Denzinger A, Schnitzler H-U (1998). Echo SPL, training experience, and experimental procedure influence the ranging performance in the big brown bat, Eptesicus fuscus. J Comp Physiol A.

[CR6] Falk B, Williams T, Aytekin M, Moss CF (2011). Adaptive behavior for texture discrimination by the free-flying big brown bat, *Eptesicus fuscus*. J Comp Physiol A.

[CR7] Firzlaff U, Schörnich S, Hoffmann S, Schuller G, Wiegrebe L (2006). A neural correlate of stochastic echo imaging. J Neurosci.

[CR8] Firzlaff U, Schuchmann M, Grunwald JE, Schuller G, Wiegrebe L (2007). Object-oriented echo perception and cortical representation in echolocating bats. PLoS Biol.

[CR9] Fremouw T, Faure PA, Casseday JH, Covey E (2005). Duration selectivity of neurons in the inferior colliculus of the big brown bat: tolerance to changes in sound level. J Neurophysiol.

[CR10] Gellermann LW (1933). Chance orders of alternating stimuli in visual discrimination experiments. Ped Sem J Gen Psychol.

[CR11] Goerlitz HR, Geberl C, Wiegrebe L (2010). Sonar detection of jittering real targets in a free-flying bat. J Acoust Soc Am.

[CR12] Griffin DR (1958). Listening in the dark: the acoustic orientation of bats and men.

[CR13] Griffin DR, Webster FA, Michael CR (1960). The echolocation of flying insects by bats. Anim Behav.

[CR14] Grossetete A, Moss CF (1998). Target flutter rate discrimination by bats using frequency-modulated sonar sounds: behavior and signal processing models. J Acoust Soc Am.

[CR15] Grunwald JE, Schornich S, Wiegrebe L (2004). Classification of natural textures in echolocation. Proc Natl Acad Sci USA.

[CR16] Halfwerk W, Jones PL, Taylor RC, Ryan MJ, Page RA (2014). Risky ripples allow bats and frogs to eavesdrop on a multisensory sexual display. Science.

[CR17] Heinrich M, Wiegrebe L (2013). Size constancy in bat biosonar? Perceptual interaction of object aperture and distance. PLoS One.

[CR18] Heinrich M, Warmbold A, Hoffmann S, Firzlaff U, Wiegrebe L (2011). The sonar aperture and its neural representation in bats. J Neurosci.

[CR19] Holderied MW, von Helversen O (2006). Binaural echo disparity’as a potential indicator of object orientation and cue for object recognition in echolocating nectar-feeding bats. J Exp Biol.

[CR20] Kwiecinski GG (2006). Phyllostomus discolor. Mamm Species.

[CR21] Lawrence BD, Simmons JA (1982). Echolocation in bats—the external ear and perception of the vertical positions of targets. Science.

[CR22] Linnenschmidt M, Wiegrebe L (2016). Sonar beam dynamics in leaf-nosed bats. Sci Rep.

[CR23] Masters WM, Jacobs SC (1989). Target detection and range resolution by the big brown bat (*Eptesicus fuscus*) using normal and time-reversed model echoes. J Comp Physiol A.

[CR24] Masters W, Raver K (1996). The degradation of distance discrimination in big brown bats (*Eptesicus fuscus*) caused by different interference signals. J Comp Physiol A.

[CR25] Menne D, Kaipf I, Wagner I, Ostwald J, Schnitzler HU (1989). Range estimation by echolocation in the bat *Eptesicus fuscus*: trading of phase versus time cues. J Acoust Soc Am.

[CR26] Miller LA (1991). Arctiid moth clicks can degrade the accuracy of range difference discrimination in echolocating big brown bats, *Eptesicus fuscus*. J Comp Physiol A.

[CR27] Moss CF, Schnitzler H-U (1989). Accuracy of target ranging in echolocating bats: acoustic information processing. J Comp Physiol A.

[CR28] Moss CF, Surlykke A (2010). Probing the natural scene by echolocation in bats. Front Behav Neurosci.

[CR29] Neuweiler G (1990). Auditory adaptations for prey capture in echolocating bats. Physiol Rev.

[CR30] Nowak RM (1994). Walker’s bats of the world.

[CR31] Roeder KD (1963). Echoes of ultrasonic pulses from flying moths. Biol Bull.

[CR32] Rother G, Schmidt U (1982). The influence of visual information on echolocation in *Phyllostomus discolor* (Chiroptera). Z Säugetierkunde.

[CR33] Roverud RC, Grinnell AD (1985). Discrimination performance and echolocation signal integration requirements for target detection and distance determination in the CF/FM bat, *Noctilio albiventris*. J Comp Physiol A.

[CR34] Roverud RC, Nitsche V, Neuweiler G (1991). Discrimination of wingbeat motion by bats, correlated with echolocation sound pattern. J Comp Physiol A.

[CR35] Sanderson MI, Simmons JA (2000). Neural responses to overlapping FM sounds in the inferior colliculus of echolocating bats. J Neurophysiol.

[CR36] Schmidt S, Nachtigall PE, Moore PWB (1988). Discrimination of target surface structure in the echolocating bat, *Megaderma lyra*. Animal sonar: processes and performance.

[CR37] Schmidt S (1988). Evidence for a spectral basis of texture perception in bat sonar. Nature.

[CR38] Schoernich S, Wiegrebe L (2008). Phase sensitivity in bat sonar revisited. J Comp Physiol A.

[CR39] Schondube JE, Herrera-M LG, Martínez del Rio C (2001). Diet and the evolution of digestion and renal function in phyllostomid bats. Zoology.

[CR40] Siemers BM, Schnitzler HU (2004). Echolocation signals reflect niche differentiation in five sympatric congeneric bat species. Nature.

[CR41] Simmons JA (1973). The resolution of target range by echolocating bats. J Acoust Soc Am.

[CR42] Simmons JA (1979). Perception of echo phase information in bat biosonar. Science.

[CR43] Simmons J, Lavender W, Lavender B, Doroshow C, Kiefer S, Livingston R, Scallet A, Crowley D (1974). Target structure and echo spectral discrimination by echolocating bats. Science.

[CR44] Simmons J, Kick S, Lawrence B, Hale C, Bard C, Escudie B (1983). Acuity of horizontal angle discrimination by the echolocating bat, *Eptesicus fuscus*. J Comp Physiol A.

[CR45] Simmons JA, Ferragamo M, Moss CF, Stevenson SB, Altes RA (1990). Discrimination of jittered sonar echoes by the echolocating bat, *Eptesicus fuscus*—the shape of target images in echolocation. J Comp Physiol A.

[CR46] Simmons JA, Ferragamo MJ, Sanderson MI (2003). Echo delay versus spectral cues for temporal hyperacuity in the big brown bat, *Eptesicus fuscus*. J Comp Physiol A.

[CR47] Simmons JA, Neretti N, Intrator N, Altes RA, Ferragamo MJ, Sanderson MI (2004). Delay accuracy in bat sonar is related to the reciprocal of normalized echo bandwidth, or Q. Proc Natl Acad Sci USA.

[CR48] Simmons JA, Houser D, Kloepper L, Surlykke A, Nachtigall PE, Fay RR, Popper AN (2014). Localization and classification of targets by echolocating bats and dolphins. Biosonar.

[CR49] Sum YW, Menne D (1988). Discrimination of fluttering targets by the FM-bat *Pipistrellus stenopterus*. J Comp Physiol A.

[CR50] Surlykke A, Miller LA (1985). The influence of arctiid moth clicks on bat echolocation; jamming or warning?. J Comp Physiol A.

[CR51] Vanderplank FL (1950). Air-speed/wing-tip speed ratios of insect flight. Nature.

[CR52] Weissenbacher P, Wiegrebe L (2003). Classification of virtual objects in the echolocating bat, *Megaderma lyra*. Behav Neurosci.

[CR53] Willig MR, Camilo GR, Noble SJ (1993). Dietary overlap in frugivorous and insectivorous bats from edaphic cerrado habitats of Brazil. J Mammal.

