# Supplementary material for: Flutter sensitivity in FM bats. Part I: delay modulation
Source: J Comp Physiol A Neuroethol Sens Neural Behav Physiol. 2018 Sep 22;204(11):929–39. doi: 10.1007/s00359-018-1291-z (PMC6208693; doi:10.1007/s00359-018-1291-z)
Supplement: Supplementary file 1 — Supplementary material 1 (DOCX 157 KB) [file 359_2018_1291_MOESM1_ESM.docx]

Supplementary Materials for „Flutter sensitivity in FM bats: I. Delay modulation“ by A. Leonie Baier & Lutz Wiegrebe (correspondence to: baier@orn.mpg.de)

**This file includes:** Fig. S1


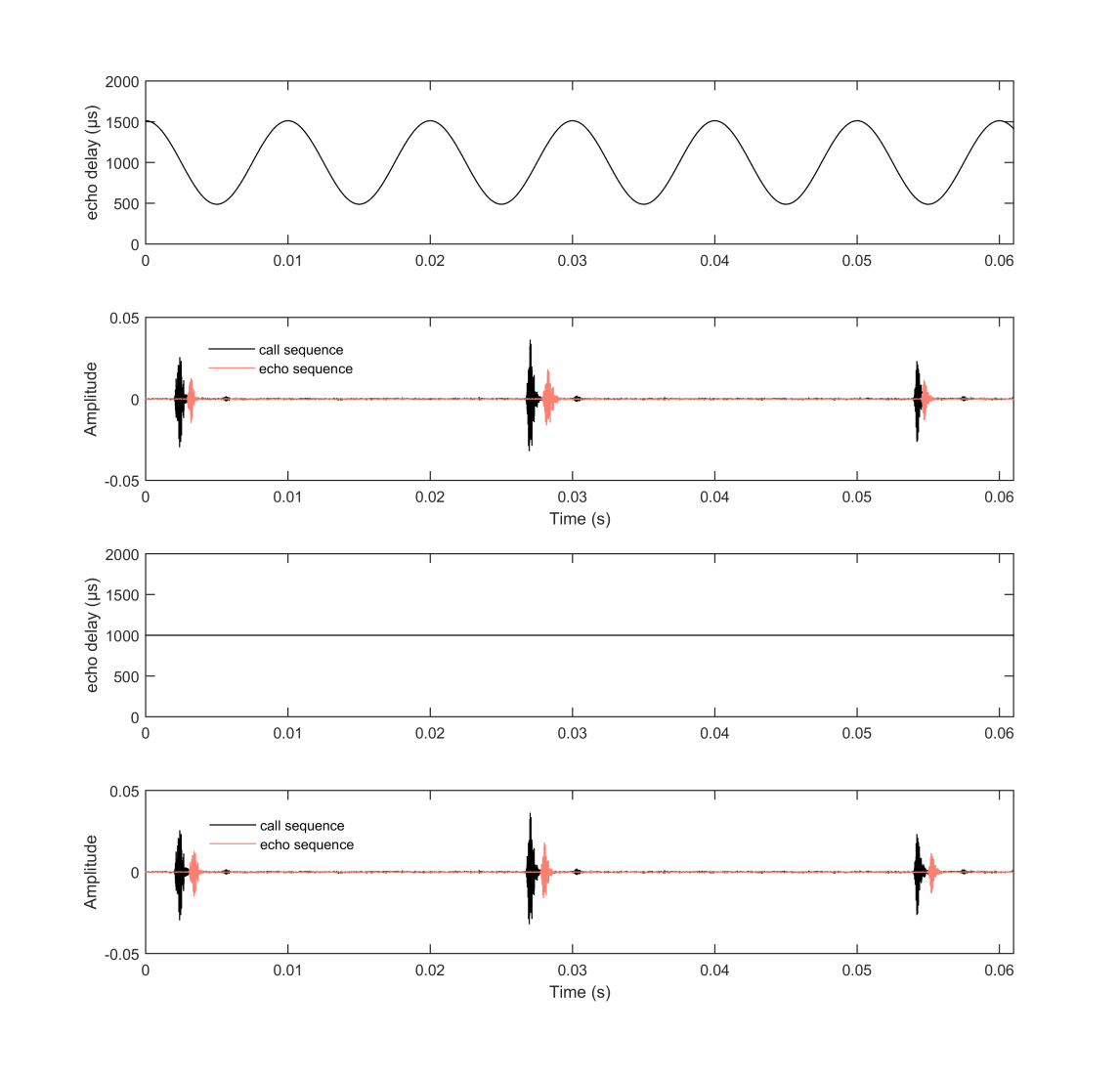


**Fig. S1** Exemplary echolocation call sequence as it was emitted by one of our bats in the experiment and illustration of the sinusoidal delay modulation. The first and third rows show how echo delay changes as a function of time, the second and fourth row show both the recorded sequence (black) and the resulting echo sequence (red). The reference echo delay was set to 1 ms for these plots to make the echo delay modulation more visible in the second row. The modulation rate in this example was 100 Hz, the modulation depth was ±512 µs in the first and second row and ±0 µs in the third and fourth row.
